# A concealed giant peritonsillolith masquerading as oropharyngeal tumor^☆^

**DOI:** 10.1016/j.bjorl.2017.09.002

**Published:** 2017-10-12

**Authors:** Boon Chye Gan, Irfan Mohamad, Norhafiza Mat Lazim

**Affiliations:** Universiti Sains Malaysia Health Campus, Department of Otorhinolaryngology-Head & Neck Surgery, Kelantan, Malaysia

## Introduction

Tonsillolith is defined as concretion of calcified materials in the crypt of palatine tonsil. Small tonsillolith is relatively common and can occur up to 10% of the population but due to the asymptomatic nature of this condition, most cases go unreported.^1^ Symptomatic patients can present with foreign body sensation, halitosis, sore throat, odynophagia and in certain cases even dysphagia or referred ear pain.^2^ Large or giant tonsillolith are fairly uncommon.^2^ Calculi found in the peritonsillar space or also known as peritonsillolith are even rarer.^3^ Peritonsillolith was first described in 1975 and since then there are only few literatures describing this entity.^4^ A giant peritonsillolith that is properly concealed by tonsil substance or mucosa may present as oropharyngeal mass that mimics tumor. We report an extremely rare case of giant peritonsillolith that underwent diagnostic tonsillectomy and surgical excision.

## Case report

A 42-year-old gentleman was referred from a periphery hospital for per oral bleeding with unilateral right tonsillar hypertrophy suspicious of oropharyngeal malignancy. He complained of multiple episodes of bleeding from the oral cavity for 3 weeks duration after brushing teeth. The bleeding was described as minimal amount of fresh blood and stopped after gargling with cold water. Upon self-examination, he noticed a swelling over the right tonsillar region with ulcer on it. There was no history of foreign body ingestion or recurrent tonsillitis. He denied odynophagia, dysphagia, neck swelling, referred otalgia or constitutional symptoms. He is a paramedic with no history of tuberculosis contact, smoking, alcohol consumption nor betel nut chewing. However, he has a strong family history of malignancy as both father and mother were diagnosed with colorectal cancer and breast cancer respectively.

**Figure 1:**
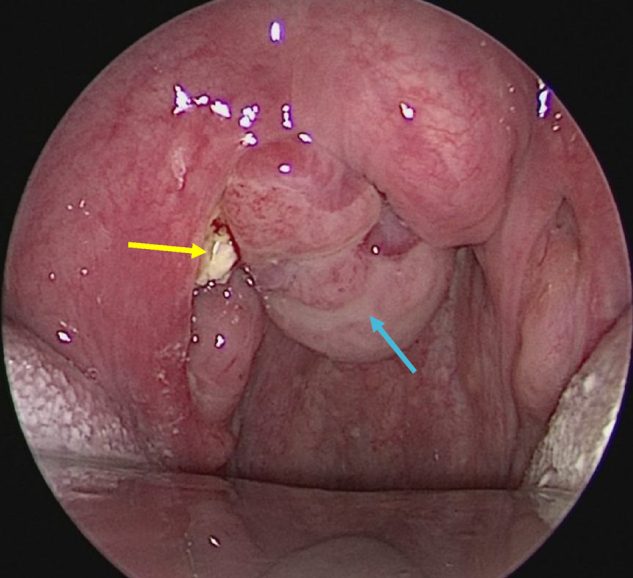
A lobulated mass was actually a huge encapsulated right peritonsillolith (blue arrow) with extension to the right peritonsillar space and small yellowish tonsillolith (yellow arrow) on the upper pole of right tonsil.

On oropharyngeal examination, bilateral tonsils were Grade 1 with smooth surface without signs to suggest acute infection nor chronic tonsillitis. There was a lobulated mass in between the right tonsil and the uvula causing slight edema and deviation of uvula to the contralateral side. There was also a small, yellowish, sloughy area at the upper pole of right tonsil (Fig. 1). Upon palpation, the mass was firm-to-hard in consistency and the yellowish area felt hard but with sharp edges. There were no palpable cervical lymph nodes. Other clinical examinations were unremarkable. A punch biopsy of the oropharyngeal mass was carried out under local anesthesia and histopathological examination (HPE) reported as inflamed granulation tissue. Full blood count, renal profile, liver function test were within normal range. Patient was then scheduled for examination under anesthesia, surgical excision and diagnostic tonsillectomy.

**Figure 2:**
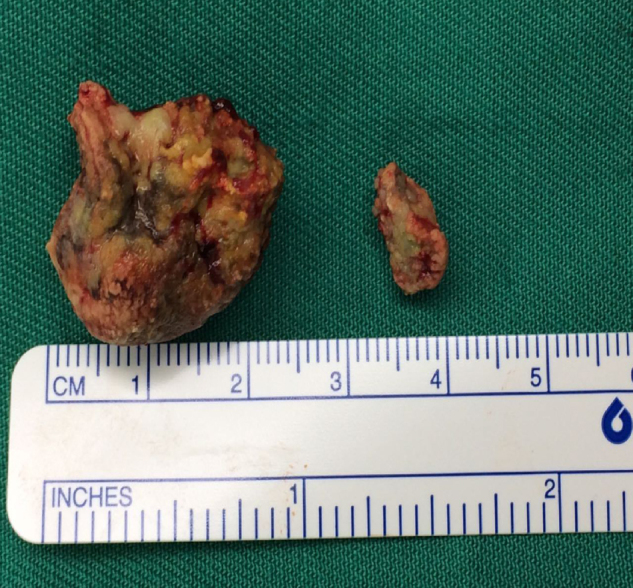
The larger round stone originated from the right peritonsillar space while the smaller oval stone was found at the upper pole of right tonsil.

Intraoperatively, upon dissecting the oropharyngeal mass, we found a large stone measuring 2.5 cm × 2.3 cm, well-concealed by a thin layer of soft tissue (Fig. 2). The stone was tracked upwards into the right peritonsillar space. A smaller stone of 1.0 cm × 0.4 cm was removed from the crypta magna of right tonsil. Tonsillectomy was then performed using dissection technique with cold instruments while hemostasis was secured with bipolar diathermy. Bilateral tonsillar fossa was normal. HPE of bilateral tonsils were reported by pathologist as reactive lymphoid hyperplasia with no evidence of malignancy. Patient was discharged and recovered well with no signs of recurrence.

## Discussion

Compared to peritonsillolith which was almost unheard of until few decades back, tonsillolith has been reported as early as 1560.^1^, ^3^, ^4^ Most tonsillolith were found in the tonsillar tissue (69.7%) followed by tonsillar fossa (21.2%) and lastly palate (9%).^2^ One of the largest tonsillolith ever reported in the literature measured up to 4.1 cm × 2.1 cm while the heaviest weighted up to 42 g.^2^ However, not much data can be found for the measurement of peritonsillolith. The largest peritonsillolith reported in literature was 2.8 cm × 2.0 cm in 2003, hence making this case to be one of the largest to date.^4^

Tonsil stone mostly contain calcium salt with occasional minerals such as phosphorus, ammonia and magnesium.^1^ This concentrated substance is not just a stone but a living biofilm as it contain infectious agents such as fungi and bacteria which is not surprising as some authors believe that tonsillolith may be a sequelae of recurrent or chronic infection.^5^, ^6^ A large stone however may come from calcification of previous peritonsillar abscess, which may be the etiopathogenesis of our case as this patient recalled having an episode of severe sore throat before without procedural history such as aspiration or incision and drainage.^7^ Other less feasible theories include stagnation of minor salivary gland flow and possible involvement of ectopic tonsillar tissue leading to stone formation.^6^

Patients with tonsillolith can present with a myriad of symptoms thus may pose as difficulty in diagnosis especially in cases where the calculi are not visible. Some patients may present with only unilateral tonsillar hypertrophy as the chief complaint. A study done on pediatric population found that unilateral tonsillar hypertrophy may just be an illusion due to depth difference of tonsillar fossa and may not indicate malignancy especially in the absence of sinister complaint or finding such as constitutional symptoms and cervical lymphadenopathy.^8^ However, in adult group, the list of differential diagnoses should always include lymphomas, squamous cell carcinomas and metastatic disease.^9^

This patient presented with chief complaint of oral bleeding which was atypical of tonsillolith. We suspect that bleeding likely occur from erosion of micro-vessels over the right tonsil by the small stone and probably aggravated by inattentive, traumatic teeth brushing. Oropharyngeal examination including intraoral palpation should be carefully performed as tonsillolith are usually diagnosed clinically because off their hard nature. Most authors agreed that radiological evaluation such as plain radiograph or computed tomography of the neck may not be necessary especially when tonsillolith can be easily palpable.^10^ Sometimes a tonsillolith can be diagnosed incidentally through a lateral neck radiograph that was taken to rule out foreign body impaction after fish bone ingestion.^11^ In cases where tumor may appear suspicious, tissue biopsy should always be taken for confirmation and in our case it was reported to be inflamed granulation tissue. If the results turned out to be a tonsillar malignancy, a different therapeutic approach will be considered. However, due to a strong family history of malignancy with clinical finding of unilateral oropharyngeal mass and history of bleeding, the diagnosis of oropharyngeal cancer must be ruled out, hence the need for examination under anesthesia, surgical excision and diagnostic tonsillectomy.^9^

## Conclusion

Management of peritonsillolith and tonsillolith is similar. In asymptomatic cases, patient can opt for watchful waiting policy or removal under local anesthesia in clinic setting by the treating otolaryngologist. However, large tonsillolith may sometimes be difficult to diagnose, especially when it is hidden behind healthy tonsil tissue. Therefore, whenever the suspicion of malignancy arises, surgical excision and diagnostic tonsillectomy should always be performed.

## Conflicts of interest

The authors declare no conflicts of interest.
